# 4313 Impaired Natural Killer Cell Function May Be Associated with Cancer-related Fatigue

**DOI:** 10.1017/cts.2020.70

**Published:** 2020-07-29

**Authors:** Jeniece Regan, Rebekah Feng, Leorey Saligan

**Affiliations:** 1 National Institutes of Health

## Abstract

OBJECTIVES/GOALS: During and after cancer treatment, cancer-related fatigue (CRF) is a debilitating symptom reported by up to 80% of cancer patients Our understanding of the pathology underlying CRF is limited. Preliminary RNA sequencing data suggest that increased levels of KIR3DL1, the natural killer cell (NK) immunoglobulin-like receptor 3DL1a, may be associated with CRF. METHODS/STUDY POPULATION: Fatigue was measured using the Functional Assessment of Chronic Illness Therapy-Fatigue (FACT-F). Functional validation of the NK cell finding was performed from whole blood obtained from fatigued and non-fatigued subjects. NK cells were isolated from freshly collected whole blood using a human NK cell isolation kit based on CD56 microbead positive selection. NK cell function was assessed using the NK cell direct cytotoxicity assay. Briefly, isolated NK cells were co-cultured in a 2:1 ratio with calcein AM-labelled K562 cells, which are NK cell-sensitive due to the very low MHCI expression. NK cell-mediated cytotoxicity was assessed with Cytation 1 Cell Imaging Multi-Mode reader. Flow cytometric protocols were used to examine NK subset differences between the fatigued and non-fatigued groups. RESULTS/ANTICIPATED RESULTS: NK cells isolated from the fatigued group exhibited decreased cytotoxicity at 12.28% compared to NK cells isolated from non-fatigued controls at a mean of 40.6% cytotoxicity. Flow cytometry analysis revealed a decrease in the CD56^dim^ CD16^bright^ population in the fatigued group (87.1% of CD56+CD4- cells) compared to the control (91.4% of CD56+CD4- cells). Furthermore, there was a decrease in NKG2A expression in mature NK cells (CD56^dim^ CD16^bright^) isolated from the fatigued group compared to the non-fatigued group. DISCUSSION/SIGNIFICANCE OF IMPACT: Results from the pilot study suggest that there was a decrease in NK cell cytotoxicity in the fatigued group. In addition, there may be a shift in NK cell subpopulations associated with fatigue. Findings from this pilot study suggest that impaired NK cell function may be associated with CRF pathogenesis.

